# ChronoMID—Cross-modal neural networks for 3-D temporal medical imaging data

**DOI:** 10.1371/journal.pone.0228962

**Published:** 2020-02-21

**Authors:** Alexander G. Rakowski, Petar Veličković, Enrico Dall’Ara, Pietro Liò

**Affiliations:** 1 Computer Laboratory, University of Cambridge, Cambridge, Cambs, England, United Kingdom; 2 Department of Oncology & Metabolism, University of Sheffield, Sheffield, South Yorkshire, England, United Kingdom; Politechnika Krakowska im Tadeusza Kosciuszki, POLAND

## Abstract

ChronoMID—neural networks for temporally-varying, hence **Chrono**, **M**edical **I**maging **D**ata—makes the novel application of cross-modal convolutional neural networks (X-CNNs) to the medical domain. In this paper, we present multiple approaches for incorporating temporal information into X-CNNs and compare their performance in a case study on the classification of abnormal bone remodelling in mice. Previous work developing medical models has predominantly focused on either spatial or temporal aspects, but rarely both. Our models seek to unify these complementary sources of information and derive insights in a bottom-up, data-driven approach. As with many medical datasets, the case study herein exhibits deep rather than wide data; we apply various techniques, including extensive regularisation, to account for this. After training on a balanced set of approximately 70000 images, two of the models—those using difference maps from known reference points—outperformed a state-of-the-art convolutional neural network baseline by over 30*pp* (> 99% vs. 68.26%) on an unseen, balanced validation set comprising around 20000 images. These models are expected to perform well with sparse data sets based on both previous findings with X-CNNs and the representations of time used, which permit arbitrarily large and irregular gaps between data points. Our results highlight the importance of identifying a suitable description of time for a problem domain, as unsuitable descriptors may not only fail to improve a model, they may in fact confound it.

## Introduction

Temporal information is especially important in the medical domain, wherein diseases develop over extended periods, treatment efficacy cannot be evaluated at the moment of administration, and temporal proximity between diseases suffered by a patient affects treatment suitability and patient recovery. In particular, studying bone remodelling (i.e. the changes over time of bone properties regulated by the activity of the bone cells) is fundamental for better understanding the effects of musculoskeletal diseases, such as osteoporosis and osteoarthritis, and to optimise the related treatments. Animal models, of mice in particular, are used for studying the effects of new treatments on bone remodelling using high-resolution medical images and microscopic computed tomography (μCT). From μCT images, proper assessment of the morphometric parameters of the bone microstructure can be used to measure the effect of diseases or interventions [1]. Furthermore, three-dimensional (3D) μCT images collected in vivo can be converted into biomechanical computational models (i.e. finite element models based on partial differential equations) for the non-invasive assessment of the bone’s mechanical properties [2]. Medical data, medical imaging data especially, contains a wealth of information which could, for example, inform diagnoses or detect early-stage diseases, yet remains largely untapped by automated processes. Due to the sheer quantities of data involved in many medical problems and the potential subtlety of informative patterns therein, the task of uncovering and extracting such patterns often falls outside the scope of human capabilities. Data-driven, automated approaches are essential for deriving usable insights from large medical datasets, such as those generated by scanners or electrocardiograms (ECGs) [3–5]. Prior work to incorporate temporal information for healthcare has focused on mining electronic patient health records for elucidating textual content or data in structured fields, thereby neglecting the temporal aspects of imaging data [6].

In this paper, we build on the successes of cross-modal convolutional neural networks [7] to incorporate both spatial and temporal information explicitly into models. We present five models, each using an alternate description of temporal information, and compare their performance against a state-of-the-art, conventional convolutional neural network (CNN) for the task of bone disease classification in mice. Our approaches involved relative timestamps, corresponding to the week the scan was taken since the study began, absolute and relative difference images, and the combination of timestamps and difference images. Two groups of mice were studied: a control group of healthy mice and a group that were treated with parathyroid hormone (PTH), which can have anabolic effects leading to accelerated bone formation when administered correctly. The data for these mice—treatment status and μCT scans of the right tibia of each mouse—were provided by Dr. Enrico Dall’Ara’s team at the University of Sheffield’s Department of Oncology and Metabolism. Our models are the first to be trained on this dataset.

Whilst different variants of recurrent neural networks (RNNs) are increasingly common for tasks involving sequences of data, they have limitations and drawbacks which we seek to overcome. Foremost, RNNs are designed to process sequential inputs, whereas medical data may have missing data points, e.g. due to patients missing appointments, or data points provided out of order, e.g. due to different administrative bodies holding patient records because people move locations or switch healthcare providers. It is preferable to make diagnoses as data becomes available rather than waiting until it has been provided in its entirety, so as to mitigate or prevent the development of pathologies. Even approaches to handling missing data for RNNs struggle to escape their sequential nature: the indicator-based approach in [8], for example, still has to provide a data point for each position in a sequence, even though that data point indicates the lack of a meaningful value at that position. Moreover, RNNs are computationally expensive, partially due to the multiple gates and units involved in memory cells, and partially due to intra-layer or backwards as well as forwards connections. These factors can lead to potentially very many parameters in a model, in turn increasing the amount of data required to train on, and thus training times. Our models improve still further on RNNs by decoupling the spatial and temporal information contained in the image set, allowing the networks to determine the relative importance of each component.

We shall begin by introducing the medical background and dataset for our case study, then move on to explain the preprocessing steps performed and justify their application in the context of the dataset and models. Thereafter, we present and contrast the models themselves, together with their performance in a classification task on the dataset and their complexity in relation to other neural network models. We conclude by discussing how model structure influences behaviour, what future challenges we foresee for medical machine learning, and where spatio-temporal, cross-modal models fit in the ever-growing sea of neural network architectures.

## Medical background

There has been growing interest in automated disease diagnosis and assessment based on machine learning (ML) models. This interest has focused on extracting features from time-series, such as using electroencephalography (EEG) data to predict epileptic attacks up to an hour in advance [9], and on various types of medical imaging [10–15]. However, these two areas of interest have typically been studied separately, thereby potentially excluding relevant data, features, and modelling techniques which could improve model performance.

In [10], Ashinsky et al sought to predict osteoarthritis using images from magnetic resonance imaging (MRI) scans, whilst both Nasser et al [12] and Montejo et al [14] applied support vector machines (SVMs), combined or in contrast with other ML techniques, to classify (non-)osteoporosis from X-rays and (non-)osteoarthritis from optical tomography-generated images respectively. Cupek et al [11] worked on the automated assessment of joint synovitis using ultrasound images, applying a set of image processing and non-neural network ML techniques to determine synovitis severity. Tiulpin et al [13], however, opted to use a modified “Siamese” CNN architecture to classify the severity of arthritis from radiographical images. This type of CNN is, like X-CNNs, a form of multi-modal neural network. For classifying photographic images of skin cancer into one of three types (benign, malignant, and non-neoplastic lesions), Esteva et al [15] likewise used CNNs, but chose to build on a pre-trained Inception v3 CNN, replacing the final classification layer with a new layer fine-tuned on their dataset.

The case study explored in this paper concerns bone remodelling and the classification of imbalanced remodelling. Remodelling encompasses bone changes due to tissue maintenance and is governed by two interacting mechanisms: formation and resorption, in turn dependent on the RANK-RANKL-OPG signalling pathway [16]. Everyday actions, such as walking, cause micro-cracks in bones to develop. If the sites of this micro-damage were simply filled in with mineralised extracellular material, the old damaged tissue would continue to be weak, compromising the mechanical integrity of the bone and risking further micro-damage accumulation. Resorption is the process of removing the damaged tissue, triggered by the signalling of osteocytes and performed by osteoclasts. Osteoblasts prevent excessive bone excavation by producing a decoy receptor, OPG, as they mature, inhibiting osteoclastic activity. The osteoblasts deposit collagen and induce mineralisation, with some of them thereafter differentiating into osteocytes embedded in the extracellular matrix. In healthy bone tissue, these processes are coupled such that the site of the micro-crack is completely repaired, but pathologies can cause imbalances that affect bone remodelling. Osteoarthritis and osteopetrosis cause excessive mineralisation and typically affect joints, due to these experiencing the greatest impacts and general wear-and-tear. This can ultimately lead to inflammation of synovial tissues, restricting movement and paining the sufferer. In contrast, diseases such as osteoporosis and osteomyelitis are caused by excessive osteoclastic activity gradually degrading bone density and reducing structural integrity, in turn making the damaged bone more vulnerable to further micro-cracks, causing additional deterioration in a vicious cycle.

One standard approach to modelling the coupled processes of formation and resorption is to use stochastic simulations based on ordinary differential equations or, when extending temporal models to spatio-temporal ones, partial differential equations [17–19]. Such models are interpretable and can use formal and probabilistic checking to verify simulation properties. However, they are reliant on biological assumptions and become increasingly difficult to understand and extend as their complexity increases, as their manual construction is dependent on a human’s capacity to comprehend and describe the system of interest [20, 21]. A resulting drawback of differential equations (DEs) and other top-down models has been that, until recently, real-world data could only be used to optimise a model’s parameters or verify its accuracy; observed data could not inform automatic changes to the fundamental structure of that model. The advent of neural ordinary DEs and their delay, stochastic, and myriad other counterparts [22, 23] not only removes the aforementioned limitation of DEs regarding automated changes to model structure, but also offers the potential to construct more powerful, parameter-efficient models than have previously been possible, with the ability to explicitly favour either processing time or error tolerances. However, this comes at the potential cost of interpretability. Neural DEs infer some or all of the dynamics of a system from the data by using one or more terms defined by neural networks; a suitable DE solver can then generate a model of the system from this and is responsible for the trade-off between computation time and accuracy. We leave the details to [22] and further discussion of combining DEs with neural networks to [23].

## Mouse data

14-week-old female C57BL/6 J (BL6) mice were purchased from Harlan Laboratories (Bicester, UK). They were housed in the University of Sheffield’s Biological Services Unit with a twelve-hour light/dark cycle at 22°C and free access to food and water. All the procedures were performed under a British Home Office project licence (PPL PCF1D350B) and in compliance with the UK Animals (Scientific Procedures) Act 1986. All surgery was performed under isoflurane anaesthesia, and all efforts were made to minimise suffering.

**Fig 1:**
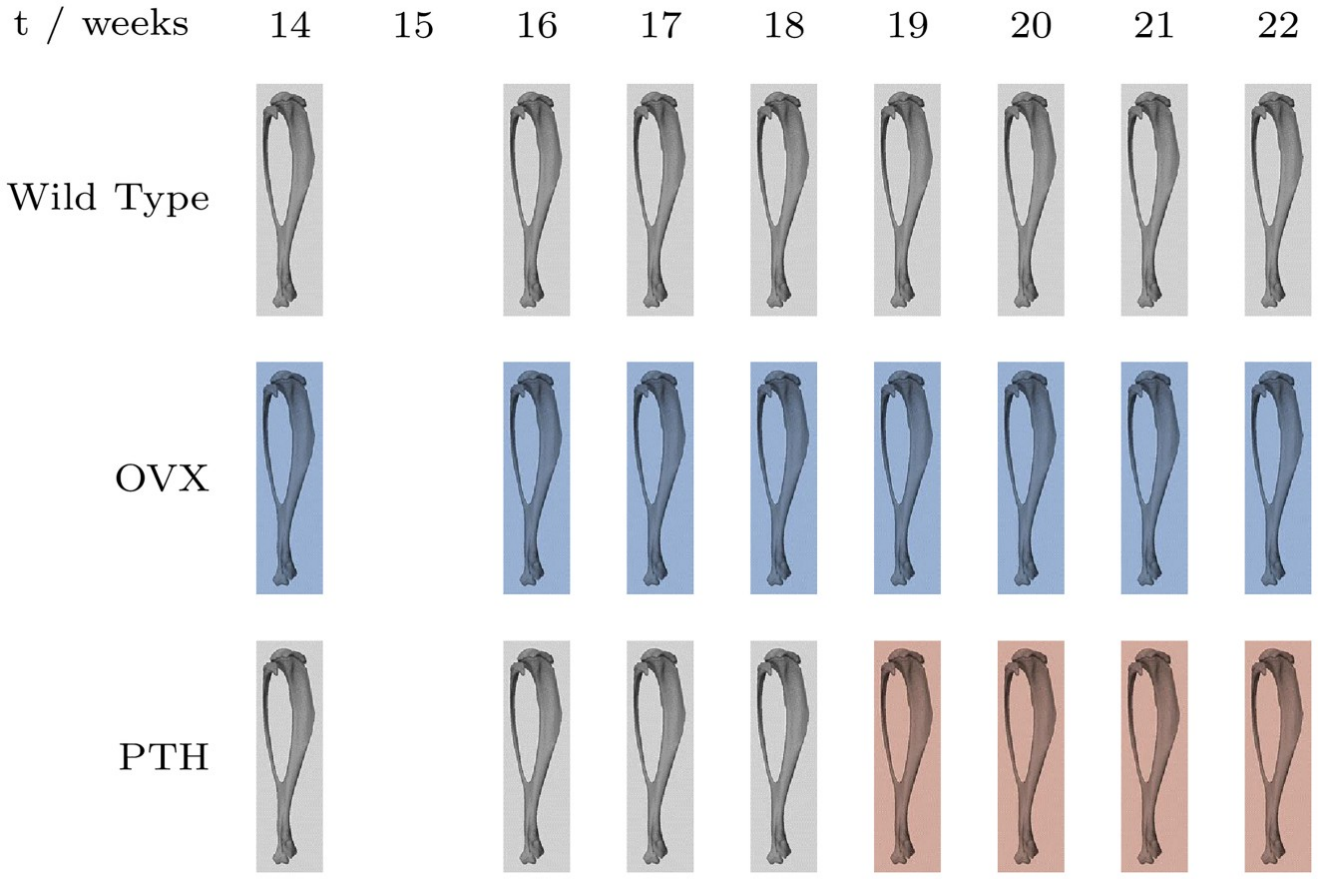
Timeline of mouse treatments. Treatment timeline of mouse groups, where *t* is the age of the mouse in weeks. Grey indicates no treatment, blue indicates ovariectomy (OVX), and red indicates treatment with PTH.

The data were μCT scans of the right tibiae of these 15 mice taken over an eight-week period, between the ages of 14 and 22 weeks, which were used to study longitudinal bone changes caused by the processes of bone resorption and formation [24]. The 15 female mice were divided into three groups (*N* = 5 per group), as shown in Fig 1, including a healthy control group and two groups with induced imbalanced bone remodelling.

The control group was referred to by the researchers as being *wild* type. The bone formation group was treated with PTH—an anabolic peptide—four weeks into the experiment, at 18 weeks of age. As such, from ages 14 to 18 weeks this group was treated identically to the control group. The bone resorption group underwent ovariectomies at 14 weeks of age, causing an œstrogen deficiency and accelerating bone resorption in weeks 14 to 22, particularly localised in the most proximal part of the bone, close to the knee. Only female mice were considered for this reason, as observed differences could be attributed to treatments rather than potentially being the result of the sex of the mice in the different groups. For our case study, we considered data from only the *wild* type and *PTH* groups.

**Fig 2:**
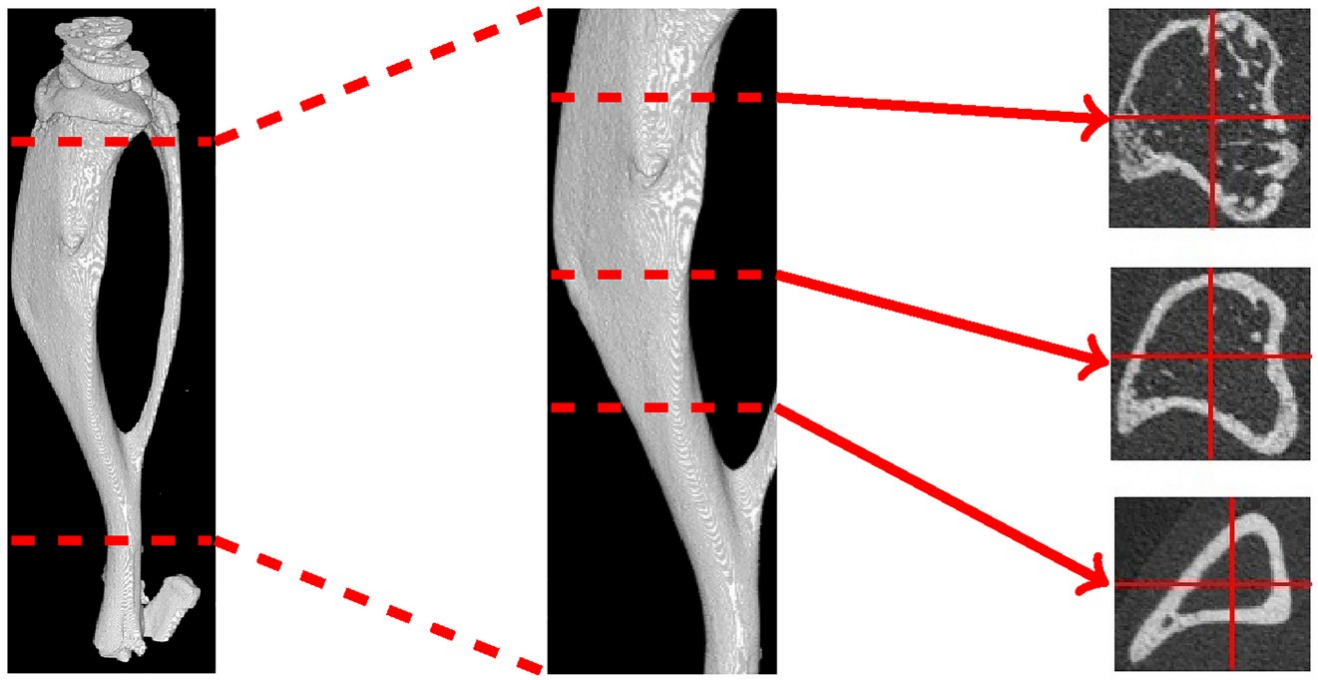
Cross-sections of a mouse tibia between the proximal and distal growth plates. Left: full tibia. Middle: section of interest between the growth plates. Right: cross-sections at three locations along the tibia.

**Fig 3:**
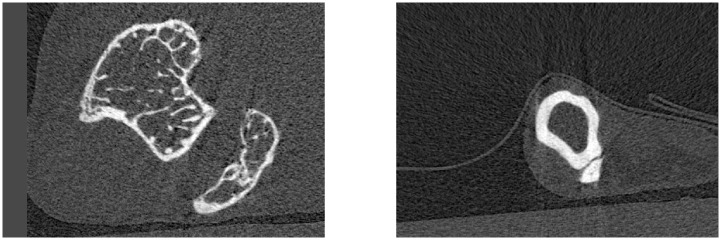
Proximal and distal image slices of a mouse tibia. μCT scan images of the right tibia of a female mouse, taken at different longitudinal positions along the bone. Left: closer to the proximal epiphysis, near the knee. Right: closer to the distal epiphysis, near the foot. Brighter areas indicate denser tissue.

In order to monitor bone changes over space and time, weekly μCT scans of the right tibia of each mouse were taken. These high-resolution scans were reconstructed in a series of slices: cross-sectional images perpendicular to the axis of the tibia and between its growth plates, as depicted in Fig 2, with a voxel size of 10μm. This resulted in around 1200-1400 slices per mouse per week, giving a total per test group of around 52000 images with a combined size of around 20GiB. Two images, taken of the same mouse at the same time, but at different longitudinal positions, are given in Fig 3.

Dr Dall’Ara’s research team performed image registration to place the images from different mice and different time points in the same reference system [25]. Due to the use of an operator crop to remove regions which did not include the tibia from the images generated by the μCT scanner, and in part due also to the mice still growing slightly, despite being considered skeletally mature, the image dimensions differ slightly from week to week and from mouse to mouse. This variation in dimensions is characterised by image heights between 400-500 pixels and widths between 500-800 pixels. All images were provided and stored in DICOM (Digital Imaging and Communications in Medicine) format. Many image processing libraries cannot accommodate variable image size, and the data were incomplete: no data were recorded for any of the mice for week two of the experiment, and one of the mice in the induced bone formation (anabolic) group was missing data for the final week. Furthermore, the data exhibited a issue common in medicine and bioinformatics: being deep rather than wide.

## Materials and methods

The data and code used in this study can be accessed via the following link in FigShare: https://doi.org/10.15131/shef.data.11764446.

### Preprocessing

**Fig 4:**
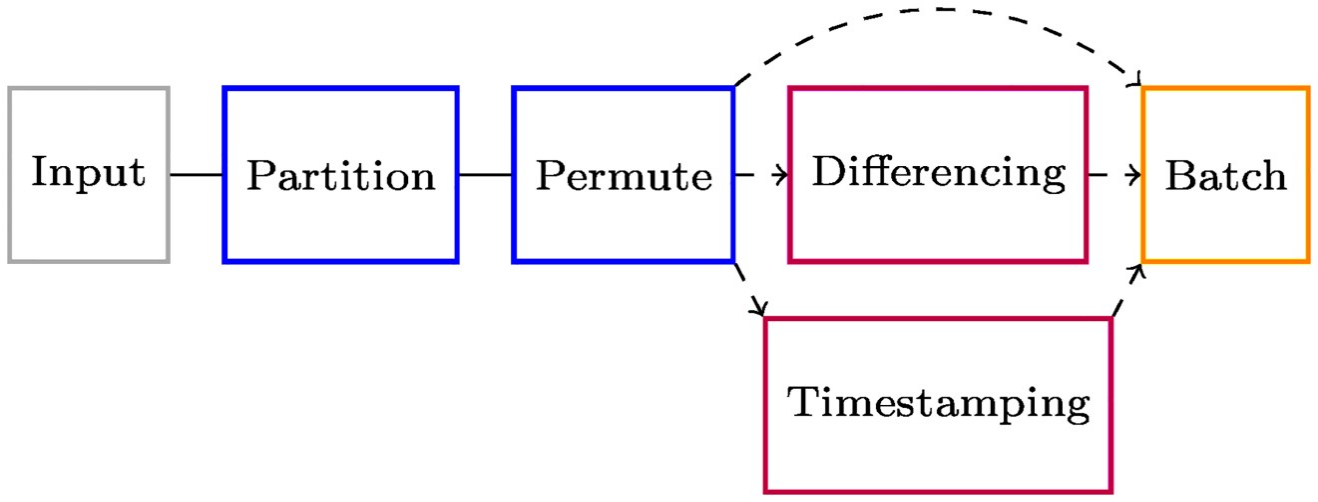
Preprocessing pipeline. Labelled images are partitioned into training, validation, and test datasets, then permuted to inject randomness. Temporal information was optionally incorporated via timestamps, difference images, or both; other descriptions of time may be substituted.

In order to both accommodate the differing image sizes and week-to-week alignment variations, and to produce the different temporal descriptions, a number of pre-processing steps were used. The pipeline of these steps is shown in Fig 4.

As most image processing libraries require images with consistent dimensions, it was necessary to convert all images to have the same width and height. As the full dataset was known in advance, it was possible to standardise the dimensions by expanding each image to the maximum height and width values observed in the dataset, which were 501 and 763 pixels respectively. This expansion process placed the image centrally and set any surrounding pixels to the minimal intensity value in that image. When the maximal image dimensions are not known in advance, images could be up- or down-sampled to fit within pre-set dimensions, either preserving relative dimensions and filling empty pixels as described, or by fitting the image to the pre-set dimensions without preserving relative sizes. This resizing process was performed once and the resulting images saved to disk to avoid the cost of resizing during each training phase.

In order to perform differencing between any two expanded images, it was first necessary to align them, as the mouse from which the images were taken would not be in exactly the same position from week to week. The alignment was performed using scikit-image’s registration method, which for a 2D image outputs a pair of numbers indicating the *x* and *y* axis translations to perform. The reference image, from an earlier timestep, underwent the specified translation for optimal alignment with the comparison image, from a later timestep, followed by pixel-wise subtraction to create the difference map. This places a comparison image and its difference map in the same frame of reference when they are provided to a model.

When each image was processed, an integer timestamp was recorded in the its meta-data, representing the week the image was taken in since the experiment’s start. Integer week numbers were recorded rather than normalised values for generalisability beyond the dataset of the case study. Timestamps normalised to the range [0, 1], for example, this would require experiment durations to be known in advance, thereby excluding later data. Such timestamps would furthermore be incompatible between datasets which, whilst otherwise comparable, had been collected over different durations.

The final stage of preprocessing was to split the dataset into three partitions for training, validation, and testing. So as to mitigate the effects of having deep rather than wide data, the unit of partitioning was a mouse-week, i.e. all images for a single mouse and a single week were assigned to the same partition. For example, “wild type” mouse 1 images in week 5 could be used for training, but then only training, in any given run. Units of mouse-week-image would risk leaking information, as a model trained on any given image would have effectively “seen” proximal images as they would be highly correlated with the training image. Using mouse-week units avoids this issue whilst permitting more granular partitioning than by mouse alone. For each run, approximately one-fifth of the mouse-weeks of data were selected at random and held out solely for testing. The remaining data was randomly permuted and partitioned as 90% for training and 10% for validation. Random permutation helps to avoid biases from repeated exposure of a network to similar training sequences and their associated gradient updates, thereby mitigating the risk of overfitting.

Due to the nature of the images, some common preprocessing steps were either undesirable or unnecessary. As the μCT scans were monochromatic, there was no opportunity to split the images into multiple colour channels or to use intensity-chromacity encodings. Separating the differing information contained in these image channels can boost model performance [7]. Thus, where applicable, chromacity and/or intensity channels should be incorporated into medical X-CNNs, applied to both the original images and the difference images. Doing so would require only minor modifications to the X-CNN approaches presented herein. In order to prevent compression artifacts, no compression was applied to the images. Instead, the neural networks effectively perform sub-sampling via pooling, in the process learning weights; sub-sampling external to the network removes such learning opportunities for the network itself. Moreover, as all images came from the same machine in the same monochromatic, DICOM format and underwent the same processing steps, no pixel normalisation was applied. Finally, data augmentation—the process of synthesising novel datasets via some perturbation or transformation of an existing dataset—was not applicable. Augmentation is fundamentally incompatible with differencing approaches as the synthesised images are generated independently from one another. This means there is no guaranteed correlation between any given reference-comparison image pair, so performing differencing on augmented images would amount to differencing randomly-selected image pairs, thereby invalidating the intent of differencing. Furthermore, augmentation is frequently used to expand small datasets and “fill in” sparse ones. For the dataset in this case study, there were large quantities of data for each mouse—around 10000 images—and as the μCT process produced image slices with high spatial proximity, adjacent images should be highly correlated and with minimal variation. As such, adjacent image slices appear to be perturbed, noisy versions of one another and week-to-week alignment variations effectively introduce small translations and rotations, all whilst retaining the temporal relationships between image slices from the same individuals at different timestamps. Given these properties, augmentation would be unlikely to benefit either this or similar datasets.

### Models

We present three different temporal descriptions and two combinations thereof, compared against a state-of-the-art CNN baseline; the architectures for these are summarised in Table 1. Note that the output layer has *n* neurons, where *n* is the number of treatment classes—two in this case study.

**Table 1:** Overview of model architectures.

	CNN, CNN timestamps	X-CNN, abs & rel	X-CNN, abs & rel, timestamps
Batch size	32		
L2 λ	0.0003		
Dropout probability	0.25		
Conv. init.	He Gaussian		
Conv. activation	ReLU		
Dense init.	He Gaussian		
Dense activation	ReLU		
Loss function	Categorical cross-entropy		
Chain 1	[3 × 3] × 16	I: [3 × 3] × 8	I: [3 × 3] × 8
		D: [3 × 3] × 8	D: [3 × 3] × 8
	[3 × 3] × 16	I: [3 × 3] × 8	I: [3 × 3] × 8
		D: [3 × 3] × 8	D: [3 × 3] × 8
	[2 × 2], stride 2	[2 × 2], stride 2	[2 × 2], stride 2
	BN	BN	BN
	Dropout	Dropout	Dropout
		I → D: [1 × 1] × 8	I → D: [1 × 1] × 8
		D → I: [1 × 1] × 8	D → I: [1 × 1] × 8
Chains 2, 3, 4	[3 × 3] × 32	I: [3 × 3] × 16	I: [3 × 3] × 16
		D: [3 × 3] × 16	D: [3 × 3] × 16
	[3 × 3] × 32	I: [3 × 3] × 16	I: [3 × 3] × 16
		D: [3 × 3] × 16	D: [3 × 3] × 16
	[2 × 2], stride 2	[2 × 2], stride 2	[2 × 2], stride 2
	BN	BN	BN
	Dropout	Dropout	Dropout
		I → D: [1 × 1] × 8	I → D: [1 × 1] × 8
		D → I: [1 × 1] × 8	D → I: [1 × 1] × 8
Chain 5	[3 × 3] × 32	I: [3 × 3] × 32	I: [3 × 3] × 32
		D: [3 × 3] × 32	D: [3 × 3] × 32
	[3 × 3] × 32	I: [3 × 3] × 32	I: [3 × 3] × 32
		D: [3 × 3] × 32	D: [3 × 3] × 32
	[2 × 2], stride 2	[2 × 2], stride 2	[2 × 2], stride 2
	BN	BN	BN
		I → D: [1 × 1] × 8	I → D: [1 × 1] × 8
		D → I: [1 × 1] × 8	D → I: [1 × 1] × 8
Dense 1	64 neurons		
	BN		
Dense 2	32 neurons		
	BN		
Output	Softmax, 2 classes		

Comparison of the model architectures, in which the following acronyms and abbreviations are used: BN = batch normalisation, abs. = absolute differencing, rel. = relative differencing, I = reference image, D = comparison image.

We chose rectified linear unit (ReLU) activation functions for their strong performance with convolutional networks [26], and He initialisations for their suitability with ReLU activations [27], as the Glorot initialisation scheme on which they are based, whilst generally applicable, was designed for sigmoidal activations [28].

**Fig 5:**
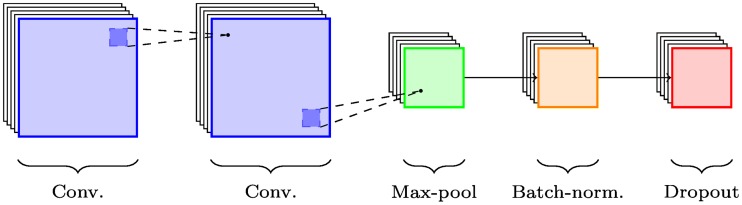
CNN chain. *Chain*—a CNN building block: 2 convolutional layers followed by max-pooling and regularised by batch normalisation and dropout.

**Fig 6:**
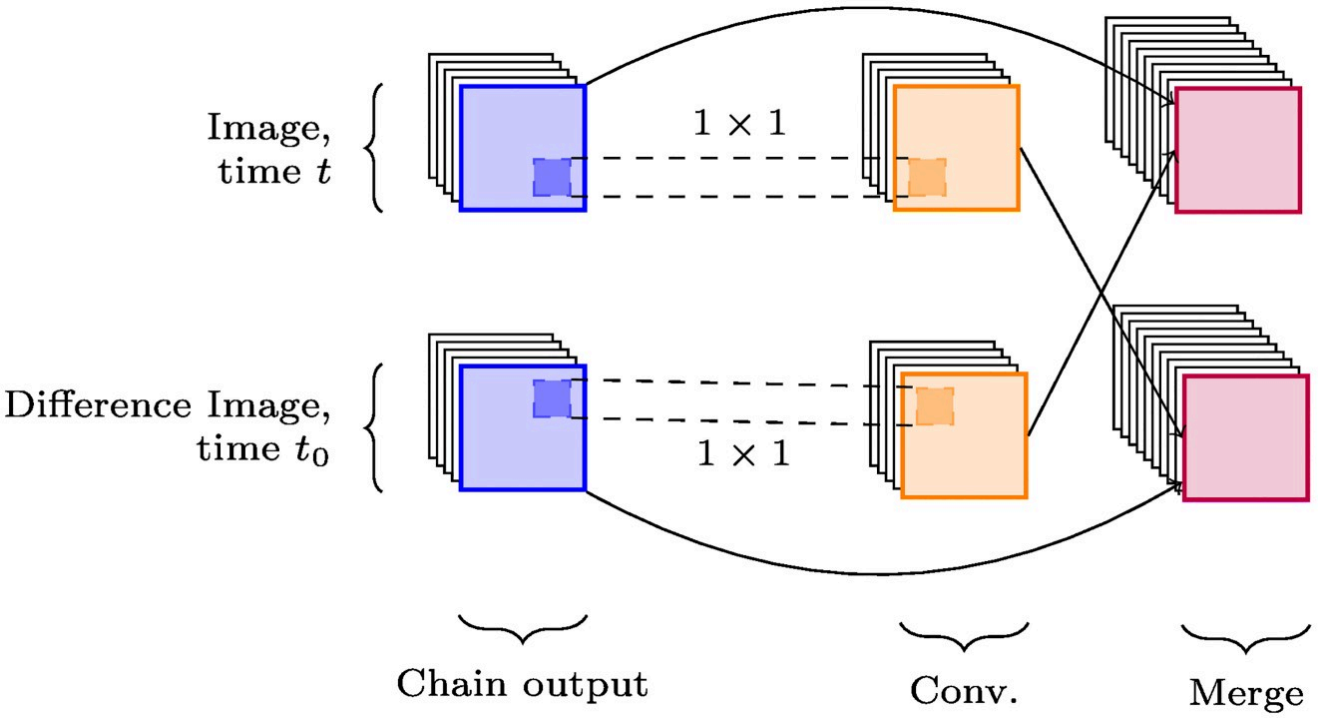
X-CNN chain. *X-chain*, featuring cross-connections between the reference and difference image channels. Cross-connections and merging may occur before or after the batch normalisation and dropout layers.

The basic building block of the convolutional part of each network is the *chain*, shown for non-cross modal networks in Fig 5 and for cross-modal ones in Fig 6. Chains consist of one or more convolutional layers, followed by pooling and any optional regularisation layers: in this case, batch normalisation and dropout.

In the fully-connected layers, it was found that batch normalisation and dropout conflicted, with the best results achieved by using batch normalisation on its own. Increasing the number of convolutional chains proved to be unsuccessful, with classification performance dropping as more chains were added. This suggests that the features of use to the network are subtle and that further compression of them into more compact features, via pooling, loses information; this further supports the decision to not downsample the images during preprocessing. Furthermore, it was found that increasing the number of neurons in the first perceptron layer to 128 or 256 did not improve the models. This was in fact advantageous, as the majority of trainable parameters in each model—around 96% for the differencing approaches—came from the connections between the convolutional and perceptron layers. Using fewer neurons in the first perceptron layer made the networks far smaller, allowing faster training.

In the case of X-CNNs, cross-connections are appended to the chain structure from Fig 5 to create what are referred to as *X-chains*, thereby enabling the exchange of feature maps between channels. These cross-connections consist of 1 × 1 convolutions of each channel and are merged via tensor concatenation. Regularisation layers such as batch normalisation may happen either before or after the cross-connection process; the impact of this is not yet fully understood.

### Baseline CNN

The baseline we used for comparison was a conventional CNN, incorporating state-of-the-art techniques.

### Timestamps

The most simple and obvious approach to incorporating time is the use of a single numeric value indicating the timestamp, which may be cardinal or ordinal. As has been discussed, timestamps were not normalised. Each timestamp was provided via a unit-sized tensor, concatenated with the output of the convolutional layers as input to the first fully-connected layer. Otherwise, this model was identical to the baseline.

### Difference images

Difference images provide an implicit description of time by performing a pixel-wise subtraction between an image at the time step of interest, *t*—the *comparison* image—and an image at a preceding time step *t*_0_—the *reference* image, should one exist:

$$\begin{array}{c} D_{ij}=I_{ij}^{\left(t\right)}-I_{ij}^{\left(t_{0}\right)} \end{array}$$ (1)

where 0 ≤ *i* < height(I), 0 ≤ *j* < width(I). Two approaches to selecting the reference image are to use images from the first recorded timestamp for each mouse or individual, referred to as *absolute* differencing, or to use images from the most recent timestamp available for each mouse or individual, referred to as *relative* differencing. These terms are used because the timestamps selected are either independent of the current timestamp, i.e. absolute, or are given relative to it. For either approach, the first timestamp’s difference images will always be of uniform intensity and equal to a minimum value; this represents the lack of preceding data. That minimum value may be decided in advance, e.g. 0, or may be the minimum value observed in each comparison image. The former requires pre-calculation or determination of a minimum value, which may not always be possible, and may introduce values in the difference image of a much larger magnitude than would be obtained in later timestamps’ difference images, placing undue emphasis on the first timestamp and the least meaningful difference image. Furthermore, it is inconsistent with the padding approach described in the image expansion process in Preprocessing. The latter approach, however, introduces global variation by allowing different initial-timestamp difference images to have different values. Ultimately we selected the latter approach, the formula for which is given below, as it is more suitable for large data sets and real-time processing.

$$\begin{array}{c} I\in M_{m\times n}\left(Z\right)\\ I_{min}=\underset{i\in [0,m),j\in [0,n)}{\text{min}}I_{i,j} \end{array}$$ (2)

Differencing faced one further difficulty, in that for each mouse there were a different number of images each week. Due to this, a one-to-one matching between reference and comparison images was not possible. As each μCT scan began at the proximal growth plate, we paired the *k*^*th*^ reference image with the *k*^*th*^ comparison image from the appropriate week, stopping when the images from either one of the weeks had been exhausted. This lead to a small proportion of images being unused in each model run.

Having both the original and difference images provides multiple processing channels, thereby enabling the use of cross-connections, making such models X-CNNs. Processing was identical for absolute and relative differencing so that any observed difference in performance could be attributed to the type of differencing used, rather than potentially being the result of different architectures or hyperparameters.

### Combining descriptions of time

It is, of course, possible to combine explicit descriptions of time, like timestamps, with implicit ones, like differencing. Providing a network with both representations allows it to determine the relative importance of each during training, thereby adapting better to the particular domain and problem. There may be situations where one temporal descriptor is more useful than another, or where one can be a deciding factor.

**Fig 7:**
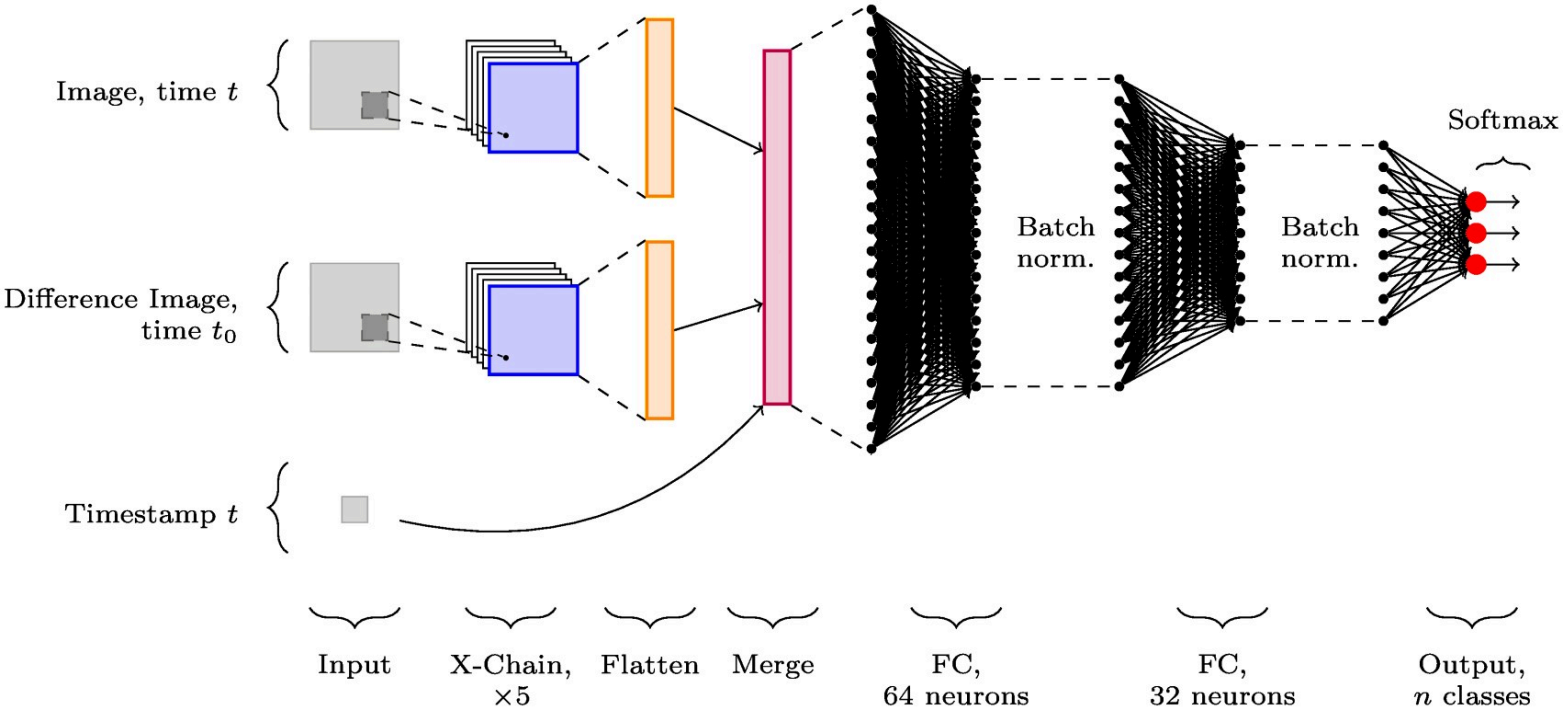
X-CNN architecture incorporating timestamps and difference images. X-CNN with channels for image data and its corresponding difference image data. Both channels are fed through five cross-connected chains, as in Fig 6. These and a timestamp channel are concatenated before being passed through fully-connected (FC) perceptron layers with batch normalisation. A softmax activation normalises the network output to the range [0, 1] for each of the *n* classes.

For combined temporal descriptions, we have three separate input channels: the original image, the difference image, and the timestamp. The image channels undergo the same processing as in Difference images before being flattened, then the tensors from all three channels are concatenated for input to the fully-connected perceptron layers; this is shown in Fig 7.

## Results

In Table 2, we present the test-set accuracies and mean training times over five and ten epochs, together with the number of parameters (both trainable and non-trainable), for each of the six models considered. The results for each model were collected from between five and ten runs, with the accuracies calculated by macro-averaging across runs and the training times calculated by micro-avergaging across runs. The variation in the number of runs per model arises from instability in the compute platform, whereby some runs only wrote out partial results; these were discarded. The two models using absolute differencing were not trained for ten epochs, due to their very high relative performance after only five epochs.

**Table 2:** Classifier accuracies and training times.

Method	Accuracy / %		Mean Time / s		Parameters
	5 Epochs	10 Epochs	5 Epochs	10 Epochs	
*CNN*	68.26 ± 7.60	60.64 ± 8.02	5889	5547	866898
*CNN, timestamps*	64.04 ± 6.12	64.09 ± 5.81	6162	6672	863914
*X-CNN, abs. diff*.	100.00 ± 0.0	–	43499	–	1643290
*X-CNN, rel. diff*.	64.59 ± 10.84	56.06 ± 11.20	45450	43909	1643290
*X-CNN, timestamps & abs. diff*.	99.84 ± 0.21	–	42884	–	1643354
*X-CNN, timestamps & rel. diff*.	61.56 ± 10.93	68.60 ± 13.67	44052	46405	1643354

Accuracy scores and per-epoch training times for the different neural network classifiers, after 5 epochs and 10 epochs of training, together with the number of parameters in each model. Accuracies were macro-averaged across runs for each model. Errors are given as standard deviations assuming Gaussian distributions.

Confusion matrices for the baseline and the top-performing model, both after five epochs of training, are provided in Table 3 for reader insight. These confusion matrices contain different total numbers of data points as they aggregate the data points from all runs for each model and the models were run a different number of times: 8 for the baseline, 5 for the X-CNN using absolute differencing.

**Table 3:** Confusion matrices for the baseline and best performing models.

	**Anabolic**	**WildType**			**Anabolic**	**WildType**
**Anabolic**	50396	37863		**Anabolic**	43740	0
**WildType**	23572	76491		**WildType**	0	54358

Left: confusion matrix for the baseline CNN after 5 epochs. Right: confusion matrix for the X-CNN with absolute differencing after 5 epochs. Note that the number of data points differs between the two matrices due to differing numbers of runs per model and different data partitioning per run.

We use accuracy, defined below, as an evaluation metric as the training, validation, and test sets were almost all well-balanced in terms of the number of images available for each treatment class. The exact splits are shown in Table 4, with standard deviations given assuming a Gaussian distribution.

$$\begin{array}{c} \text{Accuracy}=\frac{TP+TN}{TP+TN+FP+FN}\\ \text{where}\\ TP=\text{predicted}\;\text{PTH},\;\text{actually}\;\text{PTH}\\ FP=\text{predicted}\;\text{PTH},\;\text{actually}\;\text{wild-type}\\ TN=\text{predicted}\;\text{wild-type},\;\text{actually}\;\text{wild-type}\\ FN=\text{predicted}\;\text{wild-type},\;\text{actually}\;\text{PTH} \end{array}$$ (3)

**Table 4:** Data splits by use case and treatment type.

Method		“Wild” / %		PTH / %		Number of Samples	
		5 Epochs	10 Epochs	5 Epochs	10 Epochs	5 Epochs	10 Epochs
CNN	Train.	50.28 ± 1.62	50.27 ± 3.84	49.72 ± 1.62	49.73 ± 3.84	75216 ± 4368	76405 ± 6642
	Val.	50.73 ± 1.44	50.39 ± 3.85	49.27 ± 1.44	49.61 ± 3.85	8358 ± 485	8490 ± 738
	Test	46.78 ± 6.00	45.57 ± 11.51	53.22 ± 6.00	54.43 ± 11.51	23540 ± 4853	24938 ± 3814
CNN, timestamps	Train.	48.97 ± 2.95	51.79 ± 4.69	51.03 ± 2.95	48.21 ± 4.69	73958 ± 3432	76067 ± 1761
	Val.	49.06 ± 3.44	51.85 ± 4.93	50.94 ± 3.44	48.15 ± 4.93	8218 ± 382	8452 ± 196
	Test	50.32 ± 10.10	40.60 ± 16.53	49.68 ± 10.10	59.40 ± 16.53	24938 ± 3814	22595 ± 1957
X-CNN, abs. diff.	Train.	50.39 ± 1.80	−	49.61 ± 1.80	−	77179 ± 4737	−
	Val.	50.15 ± 1.78	−	49.85 ± 1.78	−	8576 ± 526	−
	Test	44.87 ± 6.39	−	55.13 ± 6.39	−	19023 ± 5263	−
X-CNN, rel. diff.	Train.	48.89 ± 1.37	49.40 ± 2.67	51.11 ± 1.37	50.60 ± 2.67	77250 ± 4713	76233 ± 7972
	Val.	48.74 ± 1.34	49.35 ± 2.62	51.26 ± 1.34	50.65 ± 2.62	8584 ± 524	8471 ± 886
	Test	52.72 ± 5.76	45.54 ± 13.73	47.28 ± 5.76	54.46 ± 13.73	20718 ± 5237	21848 ± 8858
X-CNN, t. & abs. diff.	Train.	50.85 ± 2.79	−	49.15 ± 2.79	−	76650 ± 2332	−
	Val.	51.12 ± 2.69	−	48.88 ± 2.69	−	8517 ± 259	−
	Test	42.69 ± 11.02	−	57.31 ± 11.02	−	19611 ± 2591	−
X-CNN, t. & rel. diff.	Train.	48.95 ± 3.38	49.97 ± 1.94	51.05 ± 3.38	50.03 ± 1.94	75359 ± 4188	78286 ± 4390
	Val.	48.83 ± 3.50	50.15 ± 1.86	51.17 ± 3.50	49.85 ± 1.86	8374 ± 465	8710 ± 488
	Test	51.14 ± 14.47	47.05 ± 9.24	48.86 ± 14.47	52.95 ± 9.24	22820 ± 4653	19456 ± 4878

Macro-averaged percentages of observations in the “wild type” and PTH treatment classes and the macro-averaged number of total observations per model run, for the training, validation, and test sets for each model type. Standard deviations are given for all values, assuming a normal distribution.

We provide the average training time per epoch, in addition to the number of epochs of training, as real-world training times for new models may impact decisions on which models to use in a medical setting, based on the available resources and how frequently models need to be re-trained. As training times are dependent on both the underlying hardware and the specific software implementations used, details on these can be found in S1 System Specifications.

## Discussion

The best performing models were those using absolute differencing, achieving over 99% accuracy after five epochs of training, both with and without timestamps. Absolute differencing on its own yielded the best results: 100.00% accuracy after 5 epochs. The accuracies of the baseline CNN after 5 epochs (68.26%) and the timestamped relative-differencing X-CNN after 10 epochs (68.60%) are comparable with that of Tiulpin et al’s Siamese and fine-tuned ResNet-34 CNNs [13] (66.71% and 67.49% respectively) and the polynomial-kernel SVM used by Nasser et al [12] (75.56%) on similar, bone disease-related classification tasks. They are also in line with the accuracy achieved by Esteva et al’s much more complex, parameter-intensive modified Inception v3 CNN [15] (72.1%) on a similarly sized dataset of ∼ 130K skin cancer images spanning three high-level classes. However, modern approaches are often capable of higher performance. The best model by Nasser et al—a linear-kernel SVM—achieved 95.50% accuracy and all but one of Montejo et al’s various ML techniques [14] achieved > 90% sensitivity and specificity; these were on osteoporosis and osteoarthritis classification tasks respectively. In a different domain, and focusing on temporal rather than spatial features, Güler et al applied an RNN to EEG signals to achieve 96.79% accuracy and comparably high sensitivity and specificity scores on a three-class epilepsy classification task [9]. Whilst the two best-performing ChronoMID models sit comfortably at the upper end of this range of high-performance classifiers, similar results are commonplace on datasets such as MNIST and CIFAR-10, highlighting the inherent difficulty of the medical domain and exemplifying the necessity of exploiting novel, informative, domain-specific feature descriptors.

In general, it was the case that training for longer—ten rather than five epochs—offered little to no benefit: for only one of the four models run for 10 epochs was there a noticeable performance increase (7.04*pp*), whereas two of the other models saw drops of around 7 − 9*pp*. This suggests that the models may be susceptible to overfitting, at least on a deep rather than wide dataset as in the case study, despite the extensive use of regularisation techniques. Given that performance after only five epochs was as high as 100%, this is in fact positive: powerful, useful models can be trained quickly.

The models which appeared resistant to overfitting during additional training both incorporated timestamps, suggesting that timestamps are not devoid of valuable information. However, given that the performance of these models was at best comparable to that achievable by the baseline, timestamps on their own appear to offer little benefit.

Note that if difference images could provide no information beyond that contained in the original images, we would expect that training a non-differencing model for twice as many epochs as a better-performing differencing one would allow its classification performance to approach that of the differencing model, because they would have processed the same total quantity of information. As this was not the case, we must conclude that difference images—at least with absolute differencing—contain valuable additional information.

Although relative differencing showed improvement with longer training, it performed noticeably worse than absolute differencing—between approximately 30*pp* and 45*pp* worse, depending on the number of training epochs and the inclusion or exclusion of timestamps. This is likely a consequence of the dependency range each model can represent, with relative differencing only able to capture short-range dependencies in a limited temporal region, whereas absolute differencing is capable of modelling arbitrarily long-range dependencies, although more efficiently than RNNs, which build long-range dependencies from the composition of short-range ones. For conditions like abnormal bone remodelling which, when averaged over cycles of periodic behaviour, exhibit monotonic trends in one or more metrics of interest, such as bone density and volume, long-range dependencies may best capture these trends. Relative differencing may struggle with such behaviours because each cycle is almost indistinguishable from the others, providing little discriminating information. On the other hand, it may be more suitable for conditions characterised by fluctuations around a central range of values, which would average out over longer time spans. In order to benefit from the ability to selectively model these differing dependency ranges, the appropriate differencing type should be chosen for any particular problem.

From Table 2, we see that the non-cross modal models required fewer than 900K parameters each, whilst the cross-modal ones required a little over 1.5 million each. In comparison with recent, high-performance image classification networks—5 million parameters in GoogLeNet, 23 million in Inception v3, 60 million in AlexNet, and 180 million in VGGNet [29]—a 1–1.5 million parameter network is computationally inexpensive. Two main points stand out when considering the training times in Table 2. Firstly, that the difference image-based approaches took around seven times as long to train as the non-differencing ones, likely due to a combination of disk-load speeds from having a larger-than-memory dataset and the cost of training a more complex network with around twice as many parameters. Secondly, training these networks necessitates the use of graphical processing units (GPUs) or highly parallel multi-core processing systems: whilst smaller than many high-performance models, they are not sufficiently computationally inexpensive to train on standard desktop systems.

## Conclusions

We have shown several approaches to the incorporation of temporal information for image-processing tasks, and have compared their performance on a balanced dataset comprising over 100000 data points across two treatment groups. Of five spatio-temporal models, only the two models using absolute differencing were consistently able to outperform an atemporal state-of-the-art CNN baseline, which they both did by a margin in excess of 30*pp*. Whilst the baseline appeared to suffer from overfitting after ten training epochs, the models incorporating timestamps maintained or improved their performance with more training, although they still trailed the best models by over 30*pp*.

It is evident from our results that selecting a temporal description that can capture and capitalise on the behaviour of a domain is crucial for creating performant models for problems in that domain. For our case study, wherein the treatments produced monotonic effects over extended periods of time, the ability of absolute differencing to capture long-range dependencies produced the best results. For certain medical applications this is highly advantageous: accurately determining the disease or treatment status of a patient may require as little as two scans some number of weeks apart, without the need for invasive procedures or regular appointments. Furthermore, the temporal models presented permit the use of sparse, non-sequential data sampled at irregular intervals, making them far more flexible than RNNs and 3D CNNs, which would require interpolation or an alternate approach to reconstructing sequential data and in doing so incurring a preprocessing cost. Through different techniques to the ChronoMID models, neural ODEs can also accommodate irregularly sampled data and may even produce generative models, albeit at the cost of having multiple components defined by various types of neural networks [22]. It is not clear from Chen et al’s paper how the complexity of those networks would scale to a complex dynamic system such as that of bone remodelling or to a feature-rich dataset as in the case study, where trajectories may need to be considered in terms of large vector fields rather than a single vector per time step.

Whilst we have shown the effectiveness of cross-modal neural networks for a particular temporally-varying medical dataset, our approach was kept general throughout and is applicable to a wider medical context. The models we developed could easily be extended or modified to suit particular domains or for further research into alternative temporal descriptions. Potential extensions include the use of both absolute and relative differencing, to capture both long- and short-range dependencies, the use of SVMs or RNNs either in place of or subsequent to the fully-connected layers, and the application of our models to colour images. This latter would allow investigation into whether differencing extracts additional information from not only the original image or luminance channel, but also from colour channels. In order to create less computationally-expensive and resource-intensive networks, the use of only difference images without their corresponding reference images should be explored, as they retain some amount of spatial information whilst representing temporal variations. Finally, with the fine-grained ability to choose between imposing and learning model structure afforded by the recently-introduced DiffEqFlux.jl neural DE library, we are curious about strategies for combining the explicitly temporal ChronoMID models with the implicitly temporal neural DE domain models. Whilst the most obvious approach is to use a neural DE where the dynamics are described by a ChronoMID model, we believe an iterative approach may be more insightful for biological systems where little is currently well-understood. We envisage the first step as being a pure ChronoMID or ChronoMID neural DE model used to gauge the tractability of the problem and uncover features of potential biological significance, for example by applying semantic image segmentation, as described in [30] and the citations therein. Thereafter, a series of refinement steps would gradually develop an interpretable domain model in a data-driven manner, with neural DEs modelling ever narrower aspects of the system and being replaced as the underlying biological mechanisms become well-understood.

The proposed approach to modelling temporally-varying systems has the potential for improving on the current preclinical assessment of the effects of treatments for musculoskeletal pathologies. The classification of the significant effects of a treatment can be used to optimise the dosage and time of treatment, or for combined interventions, such as providing alternating or overlapping treatments with anabolic and anti-resorptive drugs, optionally in combination with mechanical stimulation. As longitudinal experiments in mice are expensive and have ethical issues related to the usage of animals in research, longitudinal experimental designs where an animal subjected to interventions is tracked over time are becoming more popular. Such experiments dramatically reduce the number of animals in research, in line with the 3 “R”s: reduction, refinement, and replacement [31, 32]. However, these approaches create a large number of high-resolution images that need to be processed with high accuracy and reproducibility in order to evaluate the effects of interventions. In this context an automated, operator-insensitive tool such as ChronoMID is beneficial for the whole pre-clinical imaging community.

From the perspective of the longitudinal characterisation of phenotypes, we believe that there are two main challenges for the future. Firstly, if researchers wish to have broad oversight on the effects of ageing, co-mordibities, and their related interventions rather than focusing on specific tissues, organs, or systems, then it will be vital to combine data obtained from different modalities. As the data collected from different modalities are unlikely to be independent, uncovering powerful, effective ways to combine this information should result in models which are greater than the sum of their parts. Differencing goes some way towards this by using a spatial format—an image—to represent a change over time, and by supporting feature-sharing between selected modalities, cross-modal networks unlock the possibility for researchers to encode domain-specific knowledge in neural networks via carefully chosen cross-connections. The second challenge we perceive is the need for increased image resolution to capture features which are not presently available, such as osteocyte lacunae within the extracellular matrix. The availability of such high-resolution images, and the features that could be extracted from them, would require research on how to create machine learning approaches capable of accommodating the width, variety, and detail of such features in an efficient, flexible manner, with consideration for the likelihood of data points being sampled irregularly, at different rates, and with missing values.

## Supporting information

S1 System Specifications Software packages and hardware used for model generation and testing. (PDF)

S1 Appendix Program code. The code used to generate the neural network classifiers, perform batch-based data loading, etc.
(ZIP)

## References

[1] Bouxsein ML, Boyd SK, Christiansen BA, Guldberg RE, Jepsen KJ, Müller R. Guidelines for assessment of bone microstructure in rodents using micro-computed tomography. Journal of Bone and Mineral Research. 2016. 10.1002/jbmr.141 20533309

[2] Oliviero S, Giorgi M, Dall’Ara E. Validation of finite element models of the mouse tibia using digital volume correlation. Journal of the Mechanical Behaviour of Biomedical Materials. 2018;86:172–184. 10.1016/j.jmbbm.2018.06.022 29986291

[3] Lyon A, Mincholé A, Martínez JP, Rodriguez B. Computational techniques for ECG analysis and interpretation in light of their contribution to medical advances. Journal of the Royal Society, Interface. 2018;15 10.1098/rsif.2017.0821 29321268 PMC5805987

[4] Hammad M, Zhang S, Wang K. A novel two-dimensional ECG feature extraction and classification algorithm based on convolution neural network for human authentication. Future Generation Computer Systems. 2019;101:180–196. 10.1016/j.future.2019.06.008

[5] Couderc JP. A unique digital electrocardiographic repository for the development of quantitative electrocardiography and cardiac safety: the Telemetric and Holter ECG Warehouse (THEW). Journal of Electrocardiology. 2010;43(6):595–600. 10.1016/j.jelectrocard.2010.07.015 20863512 PMC2989667

[6] Choi E, Schuetz A, Stewart WF, Sun J. Using recurrent neural network models for early detection of heart failure onset. Journal of the American Medical Informatics Association. 2016;24:361–370. 10.1093/jamia/ocw112 PMC5391725 27521897

[7] Veličković P, Wang D, Lane ND, Liò P. X-CNN: Cross-modal Convolutional Neural Networks for Sparse Datasets; 2016. Available from: https://arxiv.org/abs/1610.00163.

[8] Lipton ZC, Kale D, Wetzel R. Directly Modeling Missing Data in Sequences with RNNs: Improved Classification of Clinical Time Series. In: Proceedings of the 1st Machine Learning for Healthcare Conference. vol. 56 of Proceedings of Machine Learning Research. PMLR; 2016. p. 253–270. Available from: http://proceedings.mlr.press/v56/Lipton16.html.

[9] Güler NF, Übeyli ED, Güler I. Recurrent neural networks employing Lyapunov exponents for EEG signals classification. Expert Systems with Applications. 2005;29:506–514. 10.1016/j.eswa.2005.04.011

[10] Ashinsky B, Bouhrara M, Coletta C, Lehallier B, Urish K, Lin PC, et al Predicting early symptomatic osteoarthritis in the human knee using machine learning classification of magnetic resonance images from the osteoarthritis initiative. Journal of Orthopaedic Research. 2017;. 10.1002/jor.23519 28084653 PMC5969573

[11] Cupek R, Ziębiński A. Automated assessment of joint synovitis activity from medical ultrasound and power doppler examinations using image processing and machine learning methods. Reumatologia. 2016;55:239–242. 10.5114/reum.2016.63664 PMC5149571 27994268

[12] Nasser Y, El Hassouni M, Brahim A, Toumi H, Lespessailles E, Jennane R. Diagnosis of osteoporosis disease from bone X-ray images with stacked sparse autoencoder and SVM classifier. In: 2017 International Conference on Advanced Technologies for Signal and Image Processing (ATSIP); 2017. p. 1–5.

[13] Tiulpin A, Thevenot J, Rahtu E, Lehenkari P, Saarakkala S. Automatic Knee Osteoarthritis Diagnosis from Plain Radiographs: A Deep Learning-Based Approach. In: Scientific Reports. vol. 8; 2017 p. 1727 Available from: 10.1038/s41598-018-20132-7. PMC5789045 29379060

[14] Montejo LD, Jia J, Kim HK, Netz U, Blaschke S, Müller G, et al Computer-aided diagnosis of rheumatoid arthritis with optical tomography, Part 2: image classification. Journal of Biomedical Optics. 2013;18 10.1117/1.JBO.18.7.076001 PMC3710916 23856916

[15] Esteva A, Kuprel B, Novoa RA, Ko J, Swetter SM, Blau HM, et al Dermatologist-level classification of skin cancer with deep neural networks. Nature. 2017;542:115–118. 10.1038/nature21056 28117445 PMC8382232

[16] Pivonka P, Zimak J, Smith DW, Gardiner BS, Dunstan CR, Sims NA, et al Theoretical investigation of the role of the RANK-RANKL-OPG system in bone remodeling. Journal of Theoretical Biology. 2010;262:306–316. 10.1016/j.jtbi.2009.09.021 19782692

[17] Graham JM, Ayati BP, Holstein SA, Martin JA. The Role of Osteocytes in Targeted Bone Remodeling: A Mathematical Model. PLOS ONE. 2013;8(5):1–10. 10.1371/journal.pone.0063884 PMC3661588 23717504

[18] Corte AD, Giorgio I, Scerrato D. A review of recent developments in mathematical modeling of bone remodeling. Proceedings of the Institution of Mechanical Engineers, Part H: Journal of Engineering in Medicine. 2019. 10.1177/0954411919857599 31203749

[19] Ayati BP, Edwards CM, Webb GF, Wikswo JP. A mathematical model of bone remodeling dynamics for normal bone cell populations and myeloma bone disease. Biology Direct. 2010;5:28 10.1186/1745-6150-5-28 20406449 PMC2867965

[20] Rudy SH, Brunton SL, Proctor JL, Kutz JN. Data-driven discovery of partial differential equations. Science Advances. 2017;3(4). 10.1126/sciadv.1602614 28508044 PMC5406137

[21] Huang Y, Liang H, Wu H. Identifying significant covariates for anti-HIV treatment response: Mechanism-based differential equation models and empirical semiparametric regression models. Statistics in Medicine. 2008;27(23):4722–4739. 10.1002/sim.3272 18407583 PMC2574674

[22] Chen TQ, Rubanova Y, Bettencourt J, Duvenaud DK. Neural Ordinary Differential Equations In: Bengio S, Wallach H, Larochelle H, Grauman K, Cesa-Bianchi N, Garnett R, editors. Advances in Neural Information Processing Systems 31. Curran Associates, Inc; 2018 p. 6571–6583. Available from: http://papers.nips.cc/paper/7892-neural-ordinary-differential-equations.pdf.

[23] Rackauckas C, Innes M, Ma Y, Bettencourt J, White L, Dixit V. DiffEqFlux.jl—A Julia Library for Neural Differential Equations. CoRR. 2019;abs/1902.02376.

[24] Lu Y, Boudiffa M, Dall’Ara E, Lui Y, Bellantuono I, Viceconti M. Longitudinal effects of Parathyroid Hormone treatment on morphological, densitometric and mechanical properties of mouse tibia. Journal of the Mechanical Behaviour of Biomedical Materials. 2017;75:244–251. 10.1016/j.jmbbm.2017.07.034 28756285

[25] Oliviero S, Lu Y, Viceconti M, Dall’Ara E. Effect of integration time on the morphometric, densitometric and mechanical properties of the mouse tibia. Journal of Biomechanics. 2017;65:203–211. 10.1016/j.jbiomech.2017.10.026 29126603

[26] Nair V, Hinton GE. Rectified Linear Units Improve Restricted Boltzmann Machines. In: Proceedings of the 27th International Conference on Machine Learning (ICML-10); 2010. p. 807–814. Available from: http://www.icml2010.org/papers/432.pdf.

[27] He K, Zhang X, Ren S, Sun J. Delving Deep into Rectifiers: Surpassing Human-Level Performance on ImageNet Classification. In: 2015 IEEE International Conference on Computer Vision (ICCV); 2015. p. 1026–1034.

[28] Glorot X, Bengio Y. Understanding the difficulty of training deep feedforward neural networks. In: Teh YW, Titterington M, editors. Proceedings of the Thirteenth International Conference on Artificial Intelligence and Statistics. vol. 9 of Proceedings of Machine Learning Research. Chia Laguna Resort, Sardinia, Italy: PMLR; 2010. p. 249–256. Available from: http://proceedings.mlr.press/v9/glorot10a.html.

[29] Szegedy C, Vanhoucke V, Ioffe S, Shlens J, Wojna Z. Rethinking the Inception Architecture for Computer Vision. ArXiv e-prints. 2015.

[30] Chen L, Papandreou G, Kokkinos I, Murphy K, Yuille AL. DeepLab: Semantic Image Segmentation with Deep Convolutional Nets, Atrous Convolution, and Fully Connected CRFs. IEEE Transactions on Pattern Analysis and Machine Intelligence. 2018;40(4):834–848. 10.1109/TPAMI.2017.2699184 28463186

[31] Viceconti M, Dall’Ara E. From bed to bench: How in silico medicine can help ageing research. Mechanisms of Ageing and Development. 2019;177:103–108. 10.1016/j.mad.2018.07.001 30005915

[32] Roberts BC, Giorgi M, Oliviero S, Wang N, Boudiffa M, Dall’Ara E. The longitudinal effects of ovariectomy on the morphometric, densitometric and mechanical properties in the murine tibia: A comparison between two mouse strains. Bone. 2019;127:260–270. 10.1016/j.bone.2019.06.024 31254730

