# Supplementary material for: ChronoMID—Cross-modal neural networks for 3-D temporal medical imaging data
Source: PLoS One. 2020 Feb 21;15(2):e0228962. doi: 10.1371/journal.pone.0228962 (PMC7034884; doi:10.1371/journal.pone.0228962)
Supplement: S1 System Specifications — (PDF) [file pone.0228962.s001.pdf]

# S1 System Specifications

The models considered in ChronoMID were run on Google Cloud Platform's AI Platform, with the platform and hardware specifications given in Tables 1 and 2 respectively. The models themselves, together with their batched data-loader, were written in Python. The version of Python used was 3.5 and the list of key package dependencies is given in Table 3.

**Table 1.** Google Cloud AI-Platform specifications.

| Type | Specification |
|---|---|
| Machine Type | n1-standard-4 |
| Runtime Version | 1.4 |

**Table 2.** Hardware specifications.

| **Component** | | **Specificaton** |
|---|---|---|
| CPU | Virtual Cores | 4 |
| GPU | Manufacturer | NVidia |
| | Model | Tesla K80 |
| | Memory type | GDDR5 |
| | Memory capacity | 24 GiB |
| RAM | Capacity | 15GiB |

**Table 3.** Python package specifications.

| **Package** | **Version** |
|---|---|
| google-cloud-storage | 1.23.0 |
| h5py | 2.10.0 |
| keras | 2.1.5 |
| numpy | 1.12.1 |
| pydicom | 1.3.0 |
| scikit-image | 0.15.0 |
| scipy | 0.19.0 |
| tensorflow | 1.4.1 |
| tensorflow-gpu | 1.4.1 |
